# Conference Report: WORKSHOP ON MATERIALS AND MEASUREMENTS FOR WIRELESS COMMUNICATIONS Gaithersburg, MD November 7–8, 1995

**DOI:** 10.6028/jres.101.076

**Published:** 1996

**Authors:** Terrell A. Vanderah

**Affiliations:** Ceramics Division, National Institute of Standards and Technology, Gaithersburg, MD 20899-0001

## 1. Introduction

Wireless communication technologies are expected to constitute one of the most important growth businesses in the world electronics industry during the balance of the 1990s; current market projections are on the order of 50 % per year [1]. Digital wireless communications (DWC) systems are syntheses of numerous key and supporting technologies including wireless portable telephones, cellular telephone systems, portable personal computers, data communications (“datacom”), global positioning systems (GPS), digital signal processing (DSP), ultra-large-scale integrated (ULSI) circuits, silicon and gallium arsenide microwave semiconductors, phase-locked loops (PLLs), direct signal synthesis (DSS), and active digital filtering.

Wireless communication technologies incubated in the 1970s, evolved in the 1980s, and are exploding in the 1990s. New technologies filling commercial bands above approximately 200 MHz are currently emerging at a rapid pace [2]. Although many factors helped spawn the ongoing wireless revolution [3], the successful development of applications in the 1980s can be traced to two key technical developments, one of which was AT&T’s development [4] of Ba_2_Ti_9_O_20_, the first ceramic exhibiting the unique combination of high dielectric constant, low dielectric loss, and minimal temperature dependence of the dielectric constant [5]. Ceramics based on this material can be manufactured with near-zero drift of resonant frequency with temperature and are key components (i.e., resonators) in all base stations today [5]; phase equilibria diagrams serving as “processing maps” for these materials were developed at the National Bureau of Standards (NBS) [6], now NIST. A second key event was the introduction of the network analyzer, allowing design engineers to rapidly and reliably evaluate the performance of breadboard circuits. In turn, this guided the development of techniques to accurately measure the intrinsic properties of dielectric resonator components at a wide range of frequencies. Standard methods and reference materials have not yet been developed for these types of measurements, a problem currently being addressed by NIST.

The ceramic requirements of microwave applications (e.g., filters, oscillators, resonators) are critical and represent the single largest cost and size driver of the end devices. *Every* modern commercial wireless communication and detection system in actual use or in advanced development incorporates oxide ceramics with unique electrical properties. Today, most of the new materials enabling dramatic advances in miniaturization and performance of wireless equipment are the fruit of Japanese R&D programs. Japan continues to lead in advanced electronic ceramics for the commercial wireless market. A 1990 report by then Secretary of Commerce Robert A. Mosbacher concluded that if existing trends continued, by the year 2000 the United States would lag behind Japan in most emerging electronic technologies and would trail the European community in several of them. Many of the emerging technologies referenced in this report are immediately associated with digital wireless communications technologies. A Japanese Technology Evaluation Center panel report concluded in February 1995 that

- Japan leads the United States in nearly every electronics packaging technology.
- Japan has clearly achieved a strategic advantage in electronics production and process technologies; Japanese competitors could be leading U.S. firms by as much as a decade in some electronic process technologies.
- This competitive advantage in electronics has been achieved as a consequence of developing low-cost, high-volume consumer products.
- Key factors in the success of Japan’s electronic industry are its infrastructure and the remarkable cohesiveness of vision and purpose in government and industry.
- In the foreseeable future, Japan will continue to dominate consumer electronics; however, *opportunities exist for the United States and other industrial countries to capture an increasing share of the market*.
- *No insurmountable barriers* can be identified that prevent the United States from regaining a significant share of the consumer electronics market; ample evidence indicates that the United States needs to aggressively pursue high-volume, low-cost electronic assembly.

## 2. Objectives and Description of the Workshop

The primary goal of this workshop was to gather together a relatively small number of individuals to identify, document, and prioritize key R&D needs which, if addressed, would help U.S. businesses become more competitive in the global commercial wireless market. A secondary goal was to obtain an overview of R&D needs in order to appropriately focus in-house technical work at NIST. A target group size of 50 was deliberately chosen in the hope that consensus could be reached on the prioritization of issues. The 1 1/2-day workshop was co-chaired by T. A. Vanderah (NIST), T. Negas (Trans-Tech, Inc.), W. Howng (Motorola Inc.), and R. G. Geyer (NIST). An invitational mailing list of 85 was generated by informal networking among the organizers and key individuals in industry and government. The invited group intentionally emphasized senior research managers with a broad view of technical issues as well as senior research staff with in-depth knowledge of technical issues. All invitees (62 % industry, 28 % government, 10 % academia) were given the opportunity to give a presentation. The NIST host site was chosen as relatively neutral, and an informal, interactive atmosphere was specifically encouraged in conference mailings.

Workshop literature stated the following topical emphases for the meeting: “The materials issues will focus on ceramic requirements which comprise the single largest cost and size drivers of end devices. In the area of measurements, the nature and need for “standard methods” to quantify dielectric constant, quality factor, and temperature coefficient of resonant frequency will serve as the focus.”

## 3. Content of the Workshop

The workshop attendance was 53 with the following profile: 51 % industry, 42 % government, 7 % academia. Representatives from the following companies attended:

AT&T
Bartley Machine
COMSAT
Concept Materials
Control Devices
Coors Ceramics
David Sarnoff Research Center
Ferro Corporation
GDK Products Dielectric Labs
Illinois Superconductor
MCV Technologies
Motorola
Penstock
Raytheon
Rogers Corporation
Trans-Tech

Individuals from NIST, Jet Propulsion Laboratory, Wright Patterson Air Force Laboratory, Army Research Laboratory, Naval Research Laboratory, National Science Foundation, University of Pennsylvania, and the University of Pittsburgh also attended.

The workshop was opened by the Director of NIST, Dr. Arati Prabhakar, who briefly described the explosive growth of wireless communication technologies and the related technical activities at NIST. The following oral presentations were given.

1. *Commercial Patterns and Projections for the Wireless Communications Industry* (J. Alberici, President, Trans-Tech, Inc.)
2. *Microwave Ceramics: Fit, Form, and Function* (M. D. Evans, Concept Materials, Inc.)
3. *Historical Perspective on Materials* (H. M. O’Bryan, AT&T)
4. *Current Status of Fundamental Knowledge: Structure, Chemistry, and Properties of Microwave Ceramics* (P. K. Davies, Univ. Pennsylvania)
5. *Chemical Synthesis and Processing Issues for Microwave Dielectrics* (P. Phule’, Univ. Pittsburgh)
6. *Ferrite Films by a Coat and Fire Process* (J. J. Ritter, NIST/Gaithersburg)
7. *Ferroelectric Oxide Ceramic Composites* (L. Sengupta and S. Sengupta, Army Research Laboratory)
8. *Superconductors for Wireless Communications* (J. Hodge, Illinois Superconductor)
9. *Materials and Measurement Requirements for High-Temperature Superconducting Wireless Communications Systems* (M. Nisenoff, Naval Research Laboratory)
10. *Ba_2_Ti_9_O_20_ Ceramics for Substrates* (H. M. O’Bryan, AT&T)
11. *An Assessment of “State of the Art” Electronic Packaging and Interconnections* (D. H. Knapke, Wright Patterson)
12. *The NIST Program on Electromagnetic Characterization of Materials* (C. M. Weil, NIST/Boulder)
13. *Evaluation of Ceramics using Ring Resonator Measurements at Microwave Frequencies* (A. E. Fathy, David Sarnoff Research Center)
14. *A Versatile Stripline Resonator Measurement Method Used at Rogers for Development of PWB Substrates for Wireless Communications* (G. R. Traut, Rogers Corp.)
15. *The Case for Standard Dielectric Substrates* (G. Kent, GDK Products)
16. *The User’s Perspective: Need for Ferrite/Garnet Loss Measurements* (J. Deriso, Trans-Tech, Inc.)
17. *Variable-Temperature Microwave Dielectric Properties of Isotropic and Anisotropic Materials* (R. G. Geyer, NIST, Boulder)

Some highlights from the oral presentations follow.

J. Alberici (President, Trans-Tech Inc.) gave a lively overview of the wireless communications industry, which is 93 % commercial and 7 % defense. The wide variety of devices and associated operating frequencies were described. Tremendous pressure exists to remain globally competitive by reducing costs 20 % annually. At the same time, the demands on processing control continue to increase as tolerances continue to decrease. Currently, industry must provide components with tolerances of ± 0.25 % in permittivity and temperature stability (of resonant frequency) of at least 0.25×10^−6^ °C^−1^ just to “stay in business.” Alberici described the relentless pressure to be “smaller, cheaper, and better.” Concerning the overall availability of resources for R&D, this speaker stated that if programs such as the Advanced Technology Program (ATP) at NIST and similar programs at ARPA disappear, “the effect on U.S. technology could be devastating.” In discussion that followed, the ATP program at NIST and the TRP program at ARPA were compared to the Japanese MITI program. Conference participants who had worked in Japan suggested that MITI is a strong *coordinating* influence, and that high-risk R&D is subsidized by the Japanese government. Alberici stated that investors’ demands for a certain level of return has led to a situation in which U.S. industry cannot afford high-risk R&D.

The following speaker, M. D. Evans (Concept Materials), mentioned the existence of the consortium RACE, Research for Advanced Communications in Europe, as being comparable to the Japanese MITI, and for which the United States has no counterpart. Detailed descriptions followed of each type of application, its state-of-the-art, and which materials are used. This speaker described the “enormous entry barrier,” i.e., the absence or immaturity of supporting technologies, that exists for the United States in the area of SAW (Surface Acoustic Wave) resonator technology. A list of the major players in this technology was given, some 30 Japanese and European companies, which were described as having a “strong support structure of government, academia, and industry.” The following summary of the current status of microwave materials was given:

- A variety of materials including polymers and ceramics such as ferrites, titanates, and piezoelectrics are required for base stations, terminals, and applications not yet envisioned.
- Consistent property information is needed. Scientific evaluation of process-structure-property evaluation (phase diagrams, mechanical analysis, physical property measurements) must be increased to develop more useful models for design. Work is needed to fill the gaps in property information databases.

Manufacturing methods must be improved to enhance automation capabilities. Higher product velocities, improved *in situ* measurement methods, and better processing control are needed to improve productivity and quickly respond to wireless application evolution. Key factors here are process simplicity, full-stream (including life cycle) integration, and improvements to fundamental design implementation.

- Alternative filter technologies and innovation are needed. Analysis of piezoelectric deposited structures and studies of single-crystal SAW/BAW (Surface Acoustic Wave/Bulk Acoustic Wave) architecture are needed to promote design evolution.
- Alternative packaging technologies and innovation are needed. Packaging is a critical ingredient for managing the costs of emerging microwave component technologies. Miniaturization is a continuing trend and methods of managing heat, corrosion, and costs will continue to be essential elements for growth and profitability.
- Research, development, and evaluation need to be product-focused. Manufacturing agility will be favored by spectrum dynamics (i.e., rapid changes in frequencies associated with applications). Market fluctuation and regulatory indecision can result in lost business.

P. K. Davies (U. Penn.) presented an overview of the state-of-the-art in the fundamental understanding of the structural and chemical basis of dielectric performance. An analysis of the relevant scientific literature was given that indicated no significant increase in the number of published research papers on this topic over the last 10 years; Japan continues to produce approximately 70 % of the publications; the United States, 10 %. It was pointed out that most microwave ceramic development is taking place outside the United States by Japan, Germany, Yugoslavia, and Russia, with an increasing number of active groups developing in Korea. Japan dominates in the funding of research and development of advanced microwave ceramics. In the United States, little basic research in this area receives federal support and industrial support for studies of “fundamentals” is scant. The speaker continued with a presentation of technical results on the relation of defect structural chemistry to dielectric performance. The results indicated that temperature stability (of resonant frequency) clearly has a structural basis. Correlations are beginning to accumulate for dielectric constant and temperature coefficient; however, almost nothing is understood about the chemical-structural basis for *Q* (quality factor; dielectric loss). Overall, the fundamental aspects of most microwave ceramics remain poorly understood with regards to predictive chemical control of dielectric performance.

In the discussion period scheduled after these talks a lively debate took place over the degree of leadership the United States should strive for given the ever-increasing global nature of the economy. The question was posed (and not definitively answered) as to what is a healthy level of in-house capability vs. foreign dependence. It was pointed out that customers are a primary source/driver for innovation and that they need to be close to manufacturers. In a general discussion of the role of the national labs, an opinion was offered that their impact is limited by intellectual property issues.

G. Kent (GDK Products, Dielectric Labs) described the needs and methods to establish traceability of permittivity measurements. Two major difficulties are encountered owing to the variety of materials and the variety of techniques that exist to make the measurements. Standard substrates are needed for traceability and for calibration; it was suggested that they need not meet all the demanding specifications of standard reference materials [7], but that they should be readily available and inexpensive. An approach was suggested that combines the emphasis on standard materials (National Physical Laboratory (UK) and NIST) and an emphasis on standard measurement techniques (ASTM). Standard substrates, not necessarily substrates cut from standard materials, should be mechanically flat with uniform thickness and minimum defects, exhibit minimum anisotropy in dielectric properties with permittivity values up to 300 and loss tangents less than 0.02, and have a low enough cost to make them readily available. A round-robin type approach was suggested among different laboratories using various techniques for comparative measurements.

J. Deriso (Trans-Tech) described the measurement challenges for microwave ferrites used in high volume for circulators in commercial cellular and PCS (personal communications services) radio applications. He described one customer as using 24 000 disks per week, four per device for one product alone. The manufacturers’ quality-control issues in meeting customer specifications include whether: 1) the measurement matches the customer’s geometry, 2) the measurement is conducted at or near the customer’s frequency and with the same magnetic bias, 3) the measurement can be carried out rapidly to facilitate frequent sampling, 4) the measurement can be carried out easily by a production technician, and 5) meaningful data can be obtained without resorting to exotic and highly expensive equipment (e.g., vibrating sample magnetometers).

Potential applications of high-temperature superconductors as improved frequency-selective filters for wireless communications were described by J. Hodge (Illinois Superconductor) and M. Nisenoff (Naval Research Laboratory). The promising advantages of reduced weight, volume, and electrical insertion loss, and a sharper frequency roll-off at band edges (permitting a larger number of useful channels) were described in detail. In addition, base stations could be farther apart. The major drawback and the key to cost feasibility is low-cost cryogenic technology, which continues to develop. A consensus was expressed that these materials have promise for future applications.

*Roundtable Discussion:* Prior to adjourning, approximately 2 hours were spent compiling two lists of R&D issues in the areas of materials and measurements. Considerable interactive discussion accompanied this process; points brought out by participants during this session include the following:

- In terms of general electronics technology, the 10–20 year future trends are toward multilayer architecture and elimination of discrete components.
- High-temperature superconductors have a promising future pending the evolution of cryogenic technology.
- The materials science of ceramic-metal interfaces will be increasingly important.
- U.S. industry should collaborate and share information, but anti-trust laws will be a hindrance.
- The “T” in NIST suggests that NIST may have a uniquely appropriate mission to investigate how small, highly successful entreprenurial companies operate to overcome obstacles to global competitiveness. For example, how is the volume/cost problem handled, and can lessons be learned by other U.S. industries to enhance their global competitiveness?
- Concerning the appropriate focus of federal laboratory research with regard to proprietary issues, one participant from a leading U.S. industry stated “Give us better tools to do proprietary work.”

Another participant from a leading U.S. company suggested that this workshop was a success, that the next meeting should be to specifically bring industrial players together to *share* information, and that this would be a major factor in evening the playing field with Japan: Japanese industry shares information and minimizes duplication. It was suggested that anti-trust concerns may be reduced with NIST organizing such a meeting, and that NIST staff should visit companies and document the duplication: “Management will respond when the extent of duplicated efforts is known.”

## 4. Results of the Workshop

After the workshop, the lists of R&D issues were mailed to industry-affililated participants who were asked to assign each issue between 0 and 10 points, 10 representing the highest importance and most urgent. Participants were specifically asked to disregard the issue of who should do the work and where the associated resources should come from. Twenty-one responses were received from the 27 industry-affiliated participants. This survey was intended to be qualitative and no attempt was made to statistically analyze the responses. The prioritized lists are given below. The issues are ranked in descending order of importance and urgency; in addition, the numerical values of the rankings are relative to the differences between the total points awarded to each issue (ranking 1 = highest total points).

### 4.1 Survey Results

#### Materials Issues

1 Fundamentals: understanding relationships between chemistry, crystal structure, and dielectric properties (*ε*_r_, τ_f_, *Q*)^1^
2 2 Phase diagrams of pertinent ceramic systems
2 2 Advanced ceramics; discovery of compositions with *ε*_r_ > 150, τ_f_ ≈ 0, high *Q*
2 2 Reaction kinetics during reactive sintering
3 Grain-boundaries: characterize chemistry and properties
4 Vapor pressures of volatile dopants to microwave ceramics
4 Micro-cracking: relate bulk to intrinsic properties
6 Interfaces

#### Measurements Issues

1 Standard materials for *ε*_r_, τ_f_, *Q*
1 Standard measurement techniques; update ASTM and “educate” (*ε*_r_, τ_f_, *Q*)
1 Nondestructive, customer’s geometry, variable frequency/temperature simple “assembly-line” methods to evaluate dielectric properties
2 Critically compile/evaluate database of intrinsic properties of well-characterized materials *ε*_r_, τ_f_, thermal expansion, conductivity, specific heat)
3 Evaluation of foreign measurement methods and critical evaluation of all methods
5 Relative “in situ” measurements (Go/No Go) of various properties
5 Room-temperature→300 °C anisotropic thermal expansion measurements by high-temperature x-ray diffraction
5 Non-destructive mechanical adhesion test methods
5 Surface measurements; convenient standard for variable power surface resistance

## 5. Wrap-Up and Feedback

The workshop was attended by a number of individuals from companies expressing interest in or intentions of entering the microwave ceramic market or immediately supporting markets. Recently, Coors Ceramics Electronic Products Group announced [8] the development of manufacturing process capabilities for dielectric ceramic materials for wireless technology. Interest in entering the microwave ceramics market was also expressed by workshop participants from several small companies.

Forms requesting anonymous feedback on the workshop were distributed to all participants before the meeting adjourned. Attendees were overwhelming positive (98 %) in indicating that the workshop was worth their time and that another should be held. Some specific written comments from participants follow:

- The workshop helped clarify what type of work should be done at national laboratories, industry, and at universities. A future workshop should try to address the competitiveness of the United States without compromising the competitive advantages of particular U.S. companies.
- NIST should act as a mediator to promote the sharing of information among U.S. industries. This is the only way to compete with Japan.
- NIST should provide measurement standards and methods for various chemical, physical, and electronic properties of critical components and materials.
- NIST should help industry assemble a microwave property measurement lab, produce a how-to manual in this area (“Best Practices”), and arrange training courses/seminars in this area of measurements.
- NIST should publish a monograph on materials systems.
- NIST can provide useful input to the high temperature superconductor (HTS) community by evaluating the various techniques for measurement of HTS films and substrates and proposing “guidelines” on how to evaluate them. NIST could provide “standard substrates”; i.e., substrates that have been carefully measured at NIST, to the community to use in normalizing QC (quality control) equipment. A need exists for a good technique to measure the surface resistance of unpatterned HTS films for QC purposes.
- The workshop included much discussion on ceramic materials and measurement directions; future topics should include metallization issues.
- NIST technical work should focus on generic chemistry and physics knowledge relevant to wireless communication that will help industry achieve a more rapid “proprietary” introduction of new microwave materials. Establishment of materials and test standards is very important in this regard.

## Footnotes

1 *ε*_r_ = relative permittivity, τ_f_ = temperature coefficient of resonant frequency, *Q* = quality factor, inversely related to dielectric loss.

2 Received identical point totals.
